# Harm reduction in hospitals: is it time?

**DOI:** 10.1186/1477-7517-6-19

**Published:** 2009-07-29

**Authors:** Beth S Rachlis, Thomas Kerr, Julio SG Montaner, Evan Wood

**Affiliations:** 1 BC Centre for Excellence in HIV/AIDS, St. Paul's Hospital Vancouver, Canada; 2 Dalla Lana School of Public Health, University of Toronto, Toronto, Canada; 3 Department of Medicine, University of British Columbia, Vancouver, Canada

## Abstract

Among persons who inject drugs (IDU), illicit drug use often occurs in hospitals and contributes to patient expulsion and/or high rates of leaving against medical advice (AMA) when withdrawal is inadequately managed. Resultant disruptions in medical care may increase the likelihood of several harms including drug resistance to antibiotics as well as costly readmissions and increased patient morbidity. In this context, there remains a clear need for the evaluation of harm reduction strategies versus abstinence-based strategies with respect to addressing ongoing issues related to substance use among addicted hospitalized patients. While hospitalization can be used to stabilize addicted patients as they recover from their acute illness and help them to achieve abstinence, patients unable to maintain abstinence should not be penalized for failing to do so at the expense of their health. This article describes harm reduction activities within hospitals and areas for future investigation.

## Introduction

Soft-tissue infections and other injection-related infections are among the main contributors to health service use among people who inject drugs (IDU) [1-6]. In many settings, the two most common reasons for emergency department (ED) visits relate to soft-tissue infections, and problems related directly to drug use (e.g., overdose)[1,2,4,6]. Not-surprisingly, many IDU use EDs as a regular point of care; IDU are generally less likely to use outpatient services compared to non-IDU[4] and generally face poor access to prevention programs and addiction treatment services [7-9].

As a result, IDU often present to EDs later in the course of their illness, and this in turn increases the likelihood for hospital admission [2,4,5]. Drug-related infections are often painful and may progress to more serious life- and limb-threatening conditions [10]. More complicated infections such as endocarditis require extended periods of treatment with intravenous antibiotics and thus may require even longer hospital stays.

However, IDU are more likely than other patients to discharge from hospitals against medical advice (AMA) [11,12]. A 2002 study noted that IDU were over four times more likely to leave AMA compared to non-IDU [12] and leaving AMA is a strong predictor for frequent readmission [11-13]; Moreover, repeated admissions for chronic medical problems are generally more costly for total days of stay than single, cost-intensive stays [13].

In addition to the high costs associated with increased health utilization, these findings also suggest that patients are not fully recovering from their illness the first time they are treated. Incomplete therapy or treatment failure may also increase the likelihood for drug resistance to antibiotics [11,13,14]. As such, uncovering why IDU are more likely to leave AMA is a necessary first step in order to improve health outcomes, although incidentally this may also decrease the high costs associated with elevated rates of health service utilization.

## Discussion

### Harm Reduction

While an abstinence-based approach to drug use generally requires that complete cessation from all non-prescribed drugs is a pre-requisite for effective addiction treatment [15], harm reduction emphasizes that efforts to improve health and social outcomes should begin with 'where a person is at' in terms of their drug use [16]. Strategies need to be maximized, both in terms of types of services offered and where they operate. Furthermore, abstinence-based programs are generally considered high-threshold referring to the eligibility criteria for participation in such programs and the state of 'readiness' individuals need to be in prior to entry [16,17]. Low threshold services, including needle exchange programmes (NEPs), have minimal requirements for involvement and put IDU in contact with a continuum of care even when they may not be ready to engage in abstinence-based treatment [18]. Harm reduction involves a continuous spectrum of strategies, from the promotion of safer and managed drug use to complete abstinence [15]. Harm reduction advocates and guidelines [19] suggest that strategies to reduce the high risk of disease transmission should be culturally relevant and implemented within multiple contexts, including health care facilities such as hospitals [18]. Indeed, evidence suggests that active drug use does occur in hospitals and is associated with leaving AMA [12,20].

In terms of specific strategies, methadone maintenance treatment (MMT) has been associated with reductions in the need for hospitalization and generally results in improvements in health care access [2,20]. NEPs work to reduce disease transmission by lowering the rate of syringe sharing and the number and length of time used syringes are in circulation [7,21-24]. Supervised Injecting Facilities (SIFs) have also demonstrated success in the reduction of HIV risk and other harms among IDU. At North America's first SIF, IDU are provided with sterile syringes, primary care services, and referral to addiction treatment, as well as to emergency care [25]. SIF use has been associated with increases in safer injecting practices [26,27], more rapid entry into detox programs [27] and generally increased uptake of addiction treatment [9].

### Gaps in Service Delivery

While achieving abstinence from illicit drug use is ideal, for many individuals, this may be difficult, particularly without adequate support. Health care for drug users often follows psychiatric models of care that involve the use of contracts developed for addiction management. When this contract is breached (i.e., drug use continues), the patient may be discharged back into the community with cessation of care [28]. Such approaches have potentially significant ethical implications as they may impede appropriate care for drug users [29].

Negative experiences with the medical establishment may also impede health care delivery for IDU [30]. Leaving hospital AMA predisposes individuals not only to poor health outcomes due to inadequate treatment but also to major disruptions in the patient-provider relationship [20]. Recently, our local teaching hospital generated controversy when a strict illicit drug use policy that essentially allows for 'evictions' of drug users who are unable to maintain abstinence while in hospital was proposed. While this policy is currently under review, similar guidelines are in place in most hospitals in North America. The fact that active drug use occurs in hospitals and is one reason why many IDU leave AMA raises the question that if active drug use was accommodated rather than banned in hospitals, rates of leaving AMA would decline. While incorporating harm reduction in hospitals to deal with addicted patients raises a host of ethical and well as staff and patient safety issues, such an approach has the potential to result not only in better health outcomes but reduced readmissions.

### Incorporation of harm reduction programs

Indeed, harm reduction programs have already shown success when integrated with medical care. Increased integration of low- and medium-threshold harm reduction strategies with primary and acute care has been associated with increasing the proportion of IDU who have regular health care [28].

For instance, the Dr. Peter Centre in Vancouver which provides low-threshold access to care for people living with HIV/AIDS including a high proportion of IDU offers one example where harm reduction has been successfully integrated with a medical facility. Many conventional barriers have been removed at the Centre including the need to remain drug-free. MMT and the distribution of condoms and clean needles are also provided [30]. An interdisciplinary team embraces harm reduction through the promotion of self-care and autonomy and in the spring of 2002, the nurses implemented a pilot project involving the supervision of injections in the nursing treatment room. An opiate-overdose protocol was also developed and illicit drugs including crack cocaine can be smoked in a designated area on the premises. By May 2003, staff had noted a reduced incidence of soft-tissue infections associated with use of the injecting room [31].

At the Dr. Peter Centre, participants are able to build trusting relationships with healthcare staff; such a facility offers an important solution to increase acceptability of care while reducing stigma among IDU. Importantly, the continuity of care from both nurses and doctors has shown to be an effective means for reducing injection-related complications and the need for hospital admission [28,30].

Specific harm reduction strategies including drug substitution for opioid addiction, smoking rooms for tobacco and illicit drugs, and protocols to help manage drug withdrawal symptoms have already demonstrated success in their integration into health care facilities and should continue to be fully implemented into hospitals. For example, in-patient MMT has been associated with a reduced likelihood of leaving AMA which may reflect adequate and appropriate management for opioid withdrawal [20]. Certifying a greater number of physicians who are able to prescribe buprenorphine has also already been shown to result in a reduced number of hospitalizations and risk of complications [32]. Providing patients presenting with obvious physical withdrawal with additional doses of opiates or short courses of benzodiazepines has been associated with reductions in agitation and early discharge [20].

Other strategies, while proven effective in community settings, still require further study given their potential role in reducing harm among hospital-admitted IDU. Supervised injecting areas and NEPs, in particular, could be evaluated as services that could be made accessible for hospital patients, particularly those with longer stays or in wards that are designated for dealing with addicted individuals. Ideally, the availability of these services would also help to facilitate positive patient-provider relationships.

## Conclusion

Active drug use occurs in hospitals and contributes to high rates of leaving AMA among IDU. As discussed, if active drug use was accommodated through more of a harm reduction approach rather than banned in hospitals, rates of leaving AMA would likely decline. Regardless, there remains the need for evaluation of several novel harm reduction interventions versus abstinence-based strategies with respect to addressing ongoing issues related to stigmatization and elevated rates of leaving AMA. This may lend itself to a randomized trial or perhaps it is better examined via observational data where the objective would be to evaluate whether the incorporation of a different harm reduction programs (e.g., safer injecting spaces) in hospitals results in reduced rates of patients leaving AMA and overall improvements in health outcomes for IDU who are able to access these services versus those who do not. Given the contact that many IDU have with EDs, it seems fitting that harm reduction programs should continue to expand to the hospital setting, particularly when the number of IDU being treated is high. The goal is to use hospitalization to stabilize addicted patients as they recover from their acute illness and see if they can be helped to achieve abstinence. However, patients unable to maintain abstinence should not be penalized for failing to do so at the expense of their health.

## Competing interests

The authors declare that they have no competing interests.

## Authors' contributions

EW and BR developed the concept of the manuscript. BR drafted the original version. TK, EW, and JSG assisted with revisions. All authors approved the final manuscript.
